# New evidence for positive selection helps explain the paternal age effect observed in achondroplasia

**DOI:** 10.1093/hmg/ddt260

**Published:** 2013-06-04

**Authors:** Deepali N. Shinde, Dominik P. Elmer, Peter Calabrese, Jérôme Boulanger, Norman Arnheim, Irene Tiemann-Boege

**Affiliations:** 1 Molecular and Computational Biology Program, University of Southern California, Los Angeles, CA 90089, USA; 2 Institute of Biophysics, Johannes Kepler University, Linz 4020, Austria; 3 Cell and Tissue Imaging Core, Centre National de la Recherche Scientifique, Institut Curie, Paris 75005, France

## Abstract

There are certain *de novo* germline mutations associated with genetic disorders whose mutation rates per generation are orders of magnitude higher than the genome average. Moreover, these mutations occur exclusively in the male germ line and older men have a higher probability of having an affected child than younger ones, known as the paternal age effect (PAE). The classic example of a genetic disorder exhibiting a PAE is achondroplasia, caused predominantly by a single-nucleotide substitution (c.1138G>A) in *FGFR3*. To elucidate what mechanisms might be driving the high frequency of this mutation in the male germline, we examined the spatial distribution of the c.1138G>A substitution in a testis from an 80-year-old unaffected man. Using a technology based on bead-emulsion amplification, we were able to measure mutation frequencies in 192 individual pieces of the dissected testis with a false-positive rate lower than 2.7 × 10^−6^. We observed that most mutations are clustered in a few pieces with 95% of all mutations occurring in 27% of the total testis. Using computational simulations, we rejected the model proposing an elevated mutation rate per cell division at this nucleotide site. Instead, we determined that the observed mutation distribution fits a germline selection model, where mutant spermatogonial stem cells have a proliferative advantage over unmutated cells. Combined with data on several other PAE mutations, our results support the idea that the PAE, associated with a number of Mendelian disorders, may be explained primarily by a selective mechanism.

## INTRODUCTION

For certain congenital disorders, it has been observed that the probability of having an affected offspring increases exponentially as a function of the father's age (reviewed in 1–3). This paternal age effect (PAE) was already observed a century ago for achondroplasia (ACH) (MIM# 100800), which is transmitted in a dominant fashion and is the most common form of dwarfism associated with disproportionate short stature. Extensive analyses of the birth incidence of achondroplasia showed that unaffected fathers in their fifties are 10-fold more likely to have offspring with a *de novo* ACH mutation compared with unaffected fathers in their twenties (4). Different mutations in the fibroblast growth factor receptor 3 (*FGFR3*) gene have been linked to ACH (p.gly375cys and p.gly380arg) (references within and reviewed in 5,6), but most *de novo* ACH cases (97%) are caused by a transition mutation (c.1138G>A) associated with the p.gly380arg amino acid change in the protein's transmembrane domain (7,8). This amino acid change leads to an upregulation of FGFR3 intracellular signaling (reviewed in 6,9,10). The incidence for new (sporadic) ACH cases is approximately one out of 15 000–30 000 live births (11,12), making the c.1138G>A mutation one of the highest reported mutable sites in the human genome. This is consistent with studies on sperm, where the average frequency for new ACH mutations in 117 healthy unaffected men (ages 18–80) has been estimated to be ∼1 out of 35 714 (range 1 out of 42 857 to 1 out of 2564) (13). In a much smaller study (men aged 30–65 years), the frequency ranged from 1 out of 25 000 to 1 out of 8000 (14).

ACH is not the only sporadic autosomal-dominant congenital disorder associated with a PAE. A PAE has also been reported for Apert, Crouzon/Pfeiffer, Muenke, Costello and Noonan syndromes and multiple endocrine neoplasia type 2B, among others (reviewed in 2,3). Including achondroplasia, all these disorders are associated with several characteristic features: (i) the sporadic incidence rate of the predominant disease causing mutation(s) are several orders of magnitude greater than the genome average mutation rate (1.2 × 10^−8^ nucleotides per generation or 9.5 × 10^−8^ for transitions at CpG sites) (15–18); (ii) in virtually all cases, the *de novo* mutations originate in unaffected fathers, indicating that the process is related to spermatogenesis; (iii) the incidence of the spontaneous mutation increases with paternal age (reviewed in 2–4); (iv) finally, these congenital conditions show dominant inheritance of gain-of-function point-mutations in developmentally important growth factor receptor-RAS/MAPK signaling pathway genes that might have similar phenotypic outcomes in terms of constitutive activation of these pathways (reviewed in 3 and 19).

To better understand the molecular and biological mechanisms underlying the nature of the PAE, a series of studies assessing the frequencies of causal mutations of several PAE disorders has been carried out in sperm and testes. Within the last decade, it was shown for Apert syndrome (20–24) and multiple endocrine neoplasia type 2B (MEN2B) (19), that the predominant mutations exhibit a selective advantage in the male germ line. Specifically, using a testis dissection strategy, the evidence presented on Apert syndrome and MEN2B suggests that the PAE and high mutation rate is associated with an altered regulation of spermatogonial function, in this case the self-renewing A pale spermatogonia (SrAp) (19–21). The mutations arise rarely and primarily in the adult testis. In contrast to wild-type SrAp, the mutant SrAp occasionally produce extra stem-cell lineages leading to the clonal expansion and a relative enrichment of mutant SrAp in local areas within the testis, thereby explaining the formation of mutation clusters and high mutation frequencies with ageing (19–21). The selective advantage is likely the result of changes in the growth factor receptor-RAS and related signaling pathways caused by the mutant protein (19,21,25).

It has previously been suggested that the ACH mutation in FGFR3 is driven by selection mechanisms similar to those seen for Apert mutations in FGFR2 (reviewed in 2,3), but so far the available data supporting this view are inconclusive. ACH mutation frequencies were measured in two small biopsies taken from one testis in each of two old men (81 and 85 years). For each man, the two biopsies showed a marked difference in the ACH mutation frequency (one was high and the other low). When the same experiment was carried out on two middle-aged men (65 and 66 years), no significant intra-individual difference was observed, and for each testis both biopsies had low mutation frequencies (14). These data suggest that the mechanism accounting for the PAE in Apert syndrome and MEN2B might also explain the ACH data on the biopsies of the two old men. But two small biopsies taken from each testis give only a glimpse of the mutation distribution in that organ, which is insufficient to infer what molecular and biological mechanisms are driving the increase of the ACH mutation frequency. In the Apert syndrome and MEN2B work, each testis was divided into 192 pieces and the mutation frequency was measured in each piece giving the complete spatial distribution for each testis (19–21). In order to understand what biological mechanisms might be driving the high frequency of the ACH mutation with paternal age, we used the complete testis dissection strategy (19–21) to characterize the spatial distribution of the ACH mutation in a single whole testis. We also developed a new mutation assay based on bead-emulsion amplification (BEA) (26–29) that achieves an extremely high sensitivity with a false-positive rate of <2.7 × 10^−6^. We then measured the c.1138G>A mutation frequency in 192 testis pieces from an unaffected 80-year-old donor and analyzed the data using computational modeling of spermatogonial stem-cell division schemes.

## RESULTS

### Distribution of the ACH mutation in the testis

**Figure 1.**
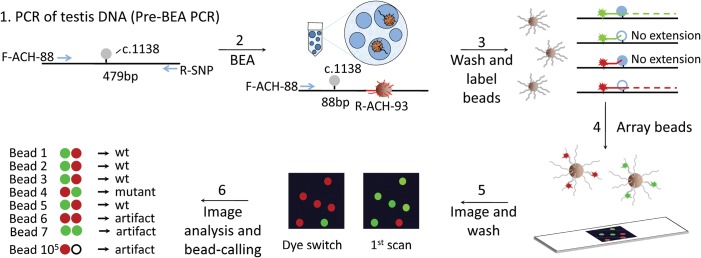
Schematic of the mutation detection. In Step 1, the DNA region with the mutation (c.1138G > A) is amplified from testis DNA with primers F-ACH-88 and R-SNP represented by blue arrows. In Step 2, a dilution of the pre-BEA PCR is used such that single amplicons are hybridized to microscopic beads coated with streptavidin and a nucleotide probe containing multiple biotins at the 5′ end and the R-ACH-93 primer sequence at the 3′ end (shown in red). The c.1138 site is amplified within the aqueous compartment of an emulsion droplet with F-ACH-88 and R-ACH-93 producing an 88 bp amplicon that stays bound to the bead. In Step 3, the beads are washed and genotyped by allele-specific extensions of fluorescent probes (colored in red and green) specific for the mutation or wild-type locus represented as empty or blue circles, respectively. In Step 4, un-extended probes are washed off and the fluorescent beads are arrayed on a slide as a monolayer. In Step 5, the array is scanned with a microscope followed by a subsequent washing, probing and imaging cycle to confirm the mutants by a dye switch. In Step 6, a series of imaging and data analysis steps are performed to estimate the mutation frequency in ∼10^5^ molecules. Different types of artifacts that are filtered out during the analysis are illustrated here.

The number of mutant ACH molecules (c.1138G>A) was assessed by a highly sensitive methodology based on the amplification of millions of single DNA molecules with BEA (27,29), followed by genotyping individual beads with previously developed methods (26,28). First, in the pre-BEA step, the initial DNA sample (1 µg of DNA of one testis piece) was amplified for 20 cycles by a high-fidelity polymerase in a conventional PCR reaction to produce FGFR3 amplicons containing the c.1138 site (Fig. 1). An aliquot of a dilution of this PCR reaction (pre-BEA PCR) was subjected to the BEA procedure. In short, individual DNA molecules from this aliquot were amplified in microscopic PCR compartments formed by an emulsion so that a magnetic bead, also within the compartment, gets coated with clonal PCR products derived from that initial single molecule. Given the microscopic volume of the BEA compartments, millions of beads can be analyzed in parallel in a single experiment. The individual beads from the BEA reaction were then genotyped with two different fluorescent dyes specific for the wild-type or the ACH mutant allele and imaged as a monolayer bead-array using an automated epifluorescent microscope, as described previously (26,28) (Fig. 1). The genotype of the beads was verified by a dye switch experiment, in which the original mutant-specific and wild type-specific dyes were switched for a second bead genotyping step. The dye switch improved the accuracy of the bead-calling, especially for rare beads (in this case mutant beads), since artifacts fluorescing in the same initial color as mutant beads could be easily identified and filtered out (Fig. 1 and Supplementary Material, Table 1). The number of mutant beads divided by the total number of wild-type and mutant beads was used to estimate the ACH mutation frequency of the original testis piece.

**Figure 2.**
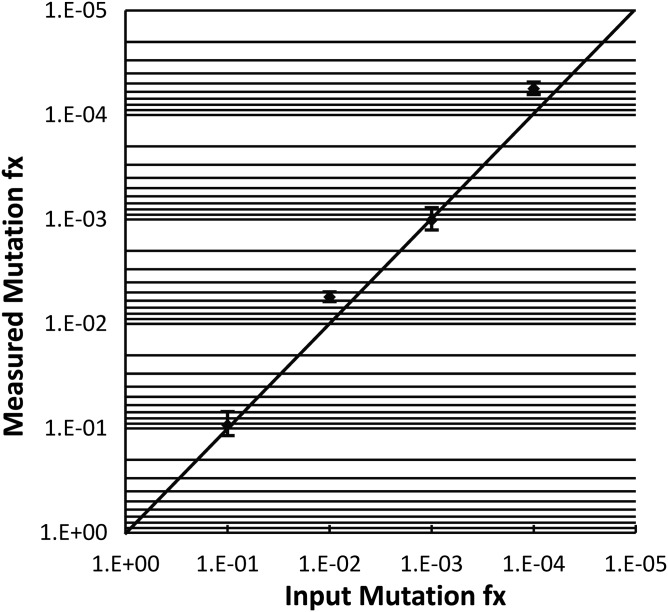
Assay validation. Mutation frequencies (Fx) estimated in a dilution series of standards derived from genomic DNA of a heterozygote ACH patient mixed at different proportions with 1 µg of normal blood DNA (1:10, 1:100, 1:1000; *n* = 3) or 330 000 plasmid DNA molecules with a wild-type FGFR3 insert plus 1 µg of *E. coli* DNA (1:10000; *n* = 2). The measured ratios shown with 95% confidence intervals match the known input ratios as represented by the hand-drawn diagonal.

We established the false-positive rate of our technology using as a proxy for the testis piece 1 μg of *E. coli* DNA spiked with 330 000 copies of a plasmid containing a wild-type ACH insert (equivalent to the amount of FGFR3 copies found in 1 µg of human DNA). We found no c.1138G>A mutations after genotyping 369 000 beads (all were wild-type), demonstrating a false-positive rate <2.7 × 10^−6^. To assess the accuracy and reproducibility of the assay, we used a series of positive controls with varying amounts of genomes with the ACH mutation spiked into wild-type DNA at mutation frequencies of 1:10, 1:100, 1:1000 and 1:10 000. We found a good correspondence between the input ratio of ACH copies to wild-type copies with respect to the actual measured ratio (Fig. 2 and Supplementary Material, Table 2). We also performed technical replicates on 18 testis pieces with low mutant counts for which a second aliquot of the initial pre-BEA PCR was analyzed (Supplementary Material, Table 3). For these pieces, the number of mutants per million genomes (pmg) was consistent and not significantly different between measurements. Two pieces with one of the highest measured frequencies (Slice 1-Piece 1 and Slice 1-Piece 3) were re-amplified from testis genomic DNA and measured again by one or multiple BEA replicates. In this case, the measurements were also reproducible (Supplementary Material, Table 3).

**Figure 3.**
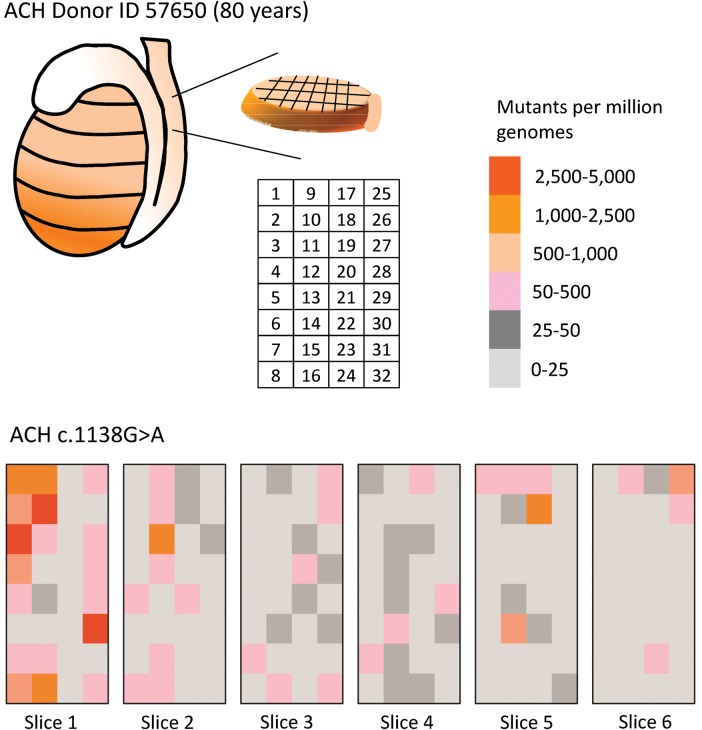
Heat map showing the distribution of the ACH mutation frequency c.1138A > G in a testis of an 80-year-old man. The testis was dissected into six slices and each slice was divided into 32 pieces (the numbering strategy of each piece in a slice is shown in the table within the figure). Each square of the heat map represents the mutation frequency measured in that piece. Data are expressed as mutants pmg.

We assayed the spatial distribution of the c.1138G>A mutation in the testis of an 80-year-old man (ID 57650). As described previously (19–21), the testis was cut into six slices and each slice was further divided into 32 pieces of approximately equal size. In Figure 3, we show the spatial distribution of the ACH mutation frequency with different colors representing the different mutation frequencies: the highest frequency is colored dark red and the lowest frequency is colored light gray. The mutation frequencies and confidence intervals for each individual testis piece are listed in Supplementary Material, Table 4. The mutations are not uniformly distributed throughout the testis but are highly clustered. In fact, there are a small number of pieces with mutation frequencies that are orders of magnitude greater than the remaining pieces. The average mutation frequency (Av) for the whole testis is 122 mutants pmg. The piece with the maximum mutation frequency (Mx) is 3072 pmg (0.31%). This piece is adjacent to a piece with no mutants. Next, we introduce several summary statistics to quantify the amount of mutation clustering. The ratio of the maximum piece frequency to the testis average frequency (Mx/Av) is 25. If the mutations were uniformly distributed and there were no clusters, this ratio would be closer to 1. Most of the pieces have low mutation frequencies: the fraction of the pieces with mutation frequencies <50 pmg (*F* < 50) is 76% (these pieces are colored light or dark gray in Fig. 3). If the mutations were uniformly distributed, none or very few of the pieces would be expected to have frequencies <50 pmg, given that the Av is 122 pmg. Finally, the fraction of the testis necessary to include 95% of the mutants (N95) is only 27%. If the mutations were uniformly distributed, one would expect this fraction to be near 95%.

### Hot spot model or selection model?

In order to understand the unexpectedly high frequency of the c.1138G>A mutation and its highly clustered distribution in the testis, we consider several computational models. We have previously proposed these models based on what is known about human germline development and maturation. More details on these models including the computer code used for the model simulations can be found in the original publications (19–21).

All the models have two phases: a growth phase and an adult phase. The growth phase models the testis from zygote formation to puberty. During the growth phase, the germ cells divide symmetrically and increase in numbers exponentially. Since the daughter cells share all the mutations of their parent cells (and possible add a few more) and remain physically close to their parent cells, mutations in the growth phase will produce mutation clusters. The growth phase is the same in all the models, but the models differ in terms of the details of the adult phase. The adult phase models the testis after puberty, when the germ cells originating in the growth phase have formed the adult self-renewing Ap spermatogonial stem cells (SrAp). We tested four models: (i) a hot spot model which predicts a uniform distribution of the mutation throughout the testis (asymmetric hot spot model); (ii) a variant of the hot spot model called the symmetric hot spot model; (iii) a selection model which predicts mutational clusters caused by the expansion of mutated SrAp (selection model) and (iv) a variant of the selection model that also incorporates cell-death at an advanced age (selection model with cell death).

#### Asymmetric hot spot model

In this model (19–21), the increased frequency of the disease mutation is due to a mutation rate per cell division at that site substantially greater than the genome average. All the divisions in the adult phase are asymmetric, yielding one daughter SrAp (self-renewal) and one differentiated daughter cell (B spermatogonium), whose descendants, after a few additional divisions, will produce sperm. In contrast to the growth phase, mutations in the adult phase will not form a mutation cluster, but will only be preserved in one SrAp cell lineage with its relatively short-lived descendant sperm and sperm precursor cells. For the asymmetric hot spot model, there is only one parameter: the mutation rate per cell division, which was inferred to be 8.1 × 10^−8^ (Table 1), thereby accounting for the high average mutation frequency of this testis (Av = 122 pmg). Note that this inferred rate is significantly greater than an inferred rate for an individual of the same age, if the site had a mutation rate equal to the generally accepted genome average mutation rate. In simulations of the asymmetric hot spot model, the spatial distribution of the mutants is relatively uniform without the extreme mutation clusters observed in the testis. This is reflected in the different summary statistics: (i) the Mx/Av ratio in the testis is 25, but in 95% of simulations this ratio is between 1.1 and 2.5; (ii) the *F* < 50 fraction in the testis is 76%, but in 95% of simulations, this fraction is 0% (in these simulations all pieces had mutation frequencies >50 pmg) and (iii) the N95 statistic in the testis is 27%, but in 95% of simulations this fraction is 95%. All three summary statistics are significantly different than measured in our data (*P* < 10^−6^) as summarized in Table 1. The conclusion is that, we strongly reject the asymmetric hot spot model. The intuition behind why this model is rejected is that there are many more cell divisions, and therefore opportunities for mutations to occur in the adult phase than in the growth phase [there are only 30 growth phase generations (30), while the adult SrAp divide every 16 days (31)]. Therefore, in simulations of this model almost all mutations occur in the adult phase and hence do not produce mutation clusters.

**Table 1.** Summary statistics of four different simulation models based on different assumptions on male germline development and maturation

Model	Mutation rate per cell division	Mx/Av^a^	*P*-value	F < 50^b^, %	*P*-value	P95^c^, %	*P*-value	Congruence of model
Observed in testis		25		76		27		
Asymmetric hot spot model	8.1 × 10^−8^	1.1–2.5	<10^−6^	0–0	<10^−6^	95–95	<10^−6^	Reject model
Symmetric hot spot model	8.1 × 10^−8^	4.1–9.0	5 × 10^−5^	27–39	<10^−6^	67–74	<10^−6^	Reject model
Selection model^d^	9.4 × 10^−11^	14.1–48	0.44	70–81	0.47	16–27	0.98	Consistent with testis data
Selection model with cell death^e^	1.8 × 10^−10^	14.2–49	0.45	70–81	0.48	16–27	0.98	Consistent with testis data

^a^Ratio of maximum mutation frequency to testis average mutation frequency.

^b^Fraction of pieces with mutation frequency <50 mutants pmg.

^c^Percentage of testis necessary to capture 95% of mutants.

^d^Selection parameter = 0.0059.

^e^Selection parameter = 0.011.

#### Symmetric hot spot model

Rejecting not just the asymmetric hot spot model, but the idea that the elevated c.1138G>A mutation frequency is due to hypermutation at this site, is a strong conclusion. So, we have also considered a model based on a different SrAp division scheme motivated by work in non-human primates (32) and mice (33). In this model, which we call the symmetric hot spot model (19), the SrAp divisions are not asymmetric in the adult phase. Rather, half of the divisions are symmetric producing two SrAp cells, and half of the divisions produce only differentiated cells that ultimately produce sperm and terminate an SrAp lineage. Both the asymmetric and the symmetric hot spot models maintain a constant number of SrAp cells and continuously produce sperm. However, the randomness of the two types of divisions in the symmetric hot spot model allows mutation clusters to randomly grow in the adult phase. If a mutated lineage divides symmetrically, there will be two mutated lineages. A series of symmetric divisions in these mutated lineages will make a mutation cluster grow, since the daughter cells remain in close physical proximity to the parent cells. But these mutation clusters can also randomly diminish and even die out, if the mutated lineages differentiate and terminate these lineages.

As shown in Table 1, the symmetric hot spot model shows slightly more evidence of mutation clustering than the hot spot model. For example, in 95% of simulations of the asymmetric hot spot model the Mx/Av ratio ranges from 1.1 to 2.5, while for the symmetric hot spot model this range is 4.1 to 9.0. However, the Mx/Av ratio observed in the testis is still significantly greater and is at 25. Similar results hold for the *F* < 50 and N95 summary statistics (Table 1). Because the clustering for the symmetric hot spot model is not as extreme as observed for the testis, we also strongly reject this model (*P* < 10^−6^).

#### Selection model

In this model, the mutated SrAp gain a new function absent in the wild-type SrAp that promotes mutation cluster formation. As in the asymmetric hot spot model, wild-type SrAp divide asymmetrically, but in this model, the mutated SrAp occasionally divides symmetrically. Since the daughter cells remain in close physical proximity to the parent cells, a series of symmetric divisions will produce a mutation cluster. The selection model (19–21) has two model parameters: the mutation rate per cell division and the selection parameter p, which is the probability that a mutant SrAp divides symmetrically. As shown in Table 1, the inferred selection parameter p is low (only 0.0059), but these rare symmetric divisions are enough to produce simulated mutation clusters similar to the ones observed in the testis. For example, in the testis, the Mx/Av ratio is 25, while in 95% of simulations of the selection model, this ratio ranges from 14.1 to 48. The results for the *F* < 50 and N95 summary statistics are also similar between the simulated and the observed data (Table 1); therefore, the selection model is consistent with the testis data.

Moreover, simulations of the model for a 25-year-old (with the inferred mutation rate per cell division taken from the 80-year-old), but the selection parameter p set to zero, so as to model a neutral site rather than a site subject to germline selection, have an average mutation frequency of 2.7 × 10^−8^. Since these simulations are for a 25-year-old, this average estimates the mutation rate per generation within a factor of 4 of the genome wide mutation rate for CpG sites (9.5 × 10^−8^) (15–18). This implies that the ACH disease mutation arises not at an elevated rate, but close to the average genome mutation rate, and that germline selection increases this mutation's frequency in the testis.

#### Selection model with cell death

Finally, we also tested a variant of the selection model that considers age-dependent cell death. The number of SrAp has been observed to decrease as men age, with most of this decrease occurring after age 60 (34,35). The details of modeling cell death are the same as in our earlier publication (19). As shown in Table 1, this model is also consistent with the ACH testis data. Furthermore, the inferred average mutation rate per generation for the selection model with cell death estimated for a 25-year-old equals 5.2 × 10^−8^, which is now within a factor of two of the estimate for transitions at CpG sites derived from genome wide data. We originally introduced cell death into the selection model to explain the observation that for MEN2B some of the oldest donors do not have mutation clusters (19). We argued that the reason that these same older testes all have substantial mutation clusters for the Apert syndrome mutation is that the selection parameter for the Apert mutation is greater than for the MEN2B mutation (19). For the hot spot models, we did not consider cell death, since incorporating cell death would make the hot spot models even less likely to produce the extreme mutation clusters observed in the testis.

## DISCUSSION

In this work, we present new data about the spatial distribution of the ACH mutation (c.1138G>A) throughout an entire testis of an 80-year-old unaffected man. We used a technology based on BEA to get a highly accurate and sensitive measurement of the c.1138G>A mutation in each of the 192 pieces of a whole testis. This method allowed us to measure mutation frequencies as low as 10^−6^, which is lower than that obtained with the only other published method to measure the ACH mutation at high sensitivity (13). The analysis of a whole testis shows that the c.1138G>A mutations are highly clustered, with a small number of testis pieces having very high mutation frequencies, while the remaining testis pieces have low frequencies. The mutation frequency of the hottest piece is 25 times higher than the average frequency of the testis. Although, only one testis was examined in this work, no exceptional mutation characteristics were observed for this testis in comparison with other age-matched testes, analyzed for the Apert and MEN2B mutations (19). Thus, it is unlikely that the observed clustered distribution of ACH mutations is exclusive to only the examined testis. In fact, consistent with our ACH data is the observation from another study (14), which revealed a difference of two orders of magnitude in the mutation frequency of c.1138G>A between two biopsies taken each from the same testis of two old donors (81 and 85 years). The observed heterogeneity of the mutation frequency between these biopsies is probably the result of the presence of similar mutation clusters we observed in this work.

### What mechanisms are driving the ACH mutation?

In spite of the high frequency of sporadic cases of achondroplasia (1/15 000–1/30 000) with new c.1138G>A transition mutations at a CpG site, an explanation evoking hypermutation at this nucleotide is not compatible with the observed clustering of the ACH mutation we detected in the testis. Both the asymmetric and symmetric hot spot models were rejected (*P* < 10^−6^). Our testis data are, however, consistent with a selection model in which mutant SrAp cells have a proliferative advantage over unmutated SrAp. All that is required is a slight chance of undergoing a symmetric division (selection model parameter = 0.0059, selection model with cell death value = 0.011). Similar clustering patterns in the testes of middle-aged and older men were also observed for Apert syndrome and MEN2B, but not in young (<21 years) testes, indicating that the clonal expansion occurs in the adult phase of spermatogenesis (19,20), and this is also likely true for the ACH mutation.

The testis we examined in this work (ID 57650) on ACH was also analyzed previously for both the Apert syndrome (c.755C>G) and MEN2B (c.2943T>C) mutations (19). For the Apert mutation, the location of clusters was concentrated in slice 6, whereas for ACH, the mutations were clustered at the opposite end, in slice 1 (Fig. 3). The independent distribution of these different PAE mutations suggests that, as would be expected, these mutations arise separately of each other. For MEN2B (19), this was one of the older testes with no clusters of note that are discussed also in the section on selection model with cell death in the Results.

### Relationship between SrAp clonal expansion and the PAE

For achondroplasia, extensive analysis of the birth incidence of affected children showed that normal fathers in their fifties are 10 times more likely to have children with a *de novo* ACH mutation than fathers in their twenties (4). Since men produce sperm throughout their lives and since each stem-cell division presents an opportunity for mutation, it is expected that there will be some increase in the germline mutation frequency as men age (1,36). However, from the twenties to the fifties, the expected increase due to the number of germline cell divisions is only 2- to 5-fold, and not as steep as observed for sporadic cases (reviewed in 2,3). In the mouse, a decrease in the efficiency of DNA repair with age has been observed (37–39). It is plausible that such a decrease also occurs in humans, thus further contributing to the increase in germline mutation frequency with age. However, any additional mutations due to the decrease in DNA repair efficiency would be spread uniformly throughout the testis and thus could not explain the observed mutation clusters in the testis. As we have shown, germline selection can explain these mutation clusters. Moreover, germline selection will also increase the frequency of the mutation super-linearly with age, thus contributing to the PAE characteristic of achondroplasia, Apert syndrome and some other conditions (4,40). This idea was further explored in a previous paper where data on the mutation frequencies of the two Apert mutations (c.755C>G and c.758C>G) were collected in over 300 sperm donors with ages ranging from 18 to 78 (23). For these mutations, a very good correlation was found between the super-linearly increasing birth data (4) and the mutation frequency measured in sperm (23), suggesting that the PAE observed in the population is correlated with a higher number of mutant sperm with age, driven by a selective process during spermatogenesis. Finally, new Apert mutations (c.755C>G) arising in *cis* to a tightly linked neutral SNP were examined in sperm of several heterozygous individuals (22). The authors suggested that the degree of allele-specific enrichment of the SNP in mutation-containing sperm was consistent with mutant spermatogonia undergoing clonal expansions (22).

Epidemiological data have shown a steep increase in the incidence of new ACH mutations with the father's age (4,40). Two papers have provided molecular data on the ACH PAE, but the results have been inconclusive. In one study with 118 sperm donors (age 22–80), a modest, but positive correlation of mutation frequency with age was found (13). However, this increase was not sufficient to explain the observed birth data (13). In a smaller study with 17 sperm donors from age 30 to 65, no significant correlation between mutation frequency and age was detected; although, mutation frequencies varied at least 3-fold (40–120 mutants pmg genomes) (14). Nonetheless, the same study (14) did find a significant correlation between mutation frequency and age in single testis biopsies (estimated to be no more than 5% of the total testis mass) from older donors (70–95 years), with differences of at least 2 orders of magnitude, but not in biopsies from middle-aged donors (53–70 years). It is unclear what would cause the discrepancy between the epidemiological, sperm and testes data.

### Possible biochemical consequences of the c.1138G>A mutation in SrAp germline cells

This study provides extensive and high-resolution data describing the clonal expansions of SrAp carrying the ACH mutation in the adult testis. This work and the earlier ACH testes biopsy data (14) provide a rationale for suggesting it is likely that this process is driven by biological mechanisms similar to those already suggested for the two most common Apert syndrome mutations in the receptor tyrosine kinase FGFR2 (19–22,24) and the MEN2B mutation in the receptor tyrosine kinase RET (19). FGFR3 is also a receptor tyrosine kinase consisting of an extracellular receptor binding domain formed by three immunoglobin-like motifs, a single transmembrane domain and a cytoplasmic tyrosine kinase domain. The binding of fibroblast growth factors (FGF), in concert with heparin sulphate proteoglycans, induce the dimerization of FGFR3 (references within and reviewed in 10,41). This dimerization further activates the intracellular catalytic domains that, through autophosphorylation, create FGFR3 interaction sites for adapter or docking proteins leading to further phosphorylation events in downstream cytoplasmic signaling cascades. Depending on the specific cell types, these include the RAS/MAPK, PI3K/AKT and STAT1 pathways among others (reviewed in 10,41). The ACH mutation (c.1138G>A) causes an amino acid change (p.gly380arg) in the transmembrane domain of FGFR3, which leads to upregulation of FGFR3 signaling that has been correlated with a compromised degradation of the mutant receptor or ligand independent activation (reviewed in 6,9,10).

Upregulation of FGRF3 signaling has different phenotypic effects depending on the tissue type. Nothing is known about how p.gly380arg affects signaling in normal SrAp; although, men with achondroplasia are fertile. Interestingly, the sterility of men with Kleinfelter syndrome may be abrogated if the man also has achondroplasia (42,43). The p.gly380arg mutation causes prolonged activation and signaling of downstream cascades including the RAS/MAPK and/or phosphoinositide-3 kinase (PI3K/AKT) pathways (reviewed in 10). Both these pathways have been shown to play a role in normal mouse spermatogonial stem cell renewal (44,45). Our results are consistent with the idea that normal FGFR3 signal transduction in SrAp cells is altered by the p.gly380arg mutation, leading to modified stem-cell self-renewal patterns that confer a proliferative advantage compared with adjacent non-mutant cells. In our simulations, a weak proliferative advantage (selection model parameter = 0.0059, selection model with cell death value = 0.011) was sufficient to drive the clonal expansion and account for the mutant clusters seen in the 80-year-old male we studied. Consistent with this idea are preliminary immunocytochemistry results on the testes of several normal older individuals that have been interpreted as possible spermatogonial microclones with altered signaling properties, but these clones have not been identified as carrying any mutation (46).

Achondroplasia is one of the most frequent genetic conditions that arise primarily as a result of new mutations at the same nucleotide site only in the male parent. Our results support the idea that the exceptionally high frequency of the c.1138A>G causative mutation is not the result of hypermutation, but rather the consequence of functional changes to SrAp carrying the mutation. Germline selection can also explain the super-linear increase in the frequency of births of affected individuals. What remains a puzzle is the discrepancy between both the sperm and birth mutation frequency data and the sperm and testes mutation frequency data. This could come about as a result of as yet unknown connections between the functional consequences of the ACH mutation in different processes of spermatogenesis and spermiogenesis. Studying other mutations with differences in activation of FGFR3 in the testis, such as hypochondroplasia (K650N) or thanatophoric dysplasia II (K650E), for which either no correlation with age or a positive correlation was detected in sperm analysis, respectively (25), could help in understanding this problem.

## MATERIALS AND METHODS

### Samples

The testis, frozen 10–12 h after death, was obtained from the National Disease Research Interchange (NDRI, Philadelphia, PA, USA) from a donor free of drug treatments that interfere with normal spermatogenesis. The sample was collected from an anonymous organ donor and complied with the ethical regulations for collection of human samples approved by the Institutional Review Board of the University of Southern California and certified as exempt (46.101 b4). Testis dissection and DNA isolation was performed as published (20,21).

### Measurement of the c.1138G>A mutations

#### Pre-BEA PCR

The number of amplifiable genomes in the DNA from each testis piece was determined by real-time PCR using primers F-ACH-88 (GAGCTGGTGGAGGCTGACGA) and R-SNP (GGTCAGCAGGGGCAGGTTGG) that included the c.1138 site. Then 1 µg of amplifiable DNA from each individual testis piece was pre-amplified for 20 cycles, which served as template DNA for the subsequent steps of emulsion PCR (BEA). Each 100 µl pre-BEA PCR reaction included 1× Phusion HF buffer (NEB), 0.2 mm each dNTP (Roche Applied Science), 2U of Phusion Hot start DNA polymerase (NEB) and 0.2 µm of each primer F-ACH-88 and R-SNP. The reactions were held at 98°C for 2 min followed by 20 cycles of 98°C for 15 s, 68°C for 10 s and 72°C for 30 s and a final extension step at 72°C for 2 min.

#### Bead-emulsion amplification

This procedure follows exactly the protocols described previously with small differences in the primers and PCR conditions used (26,28). Specifically, 1.5 µl of a 1:100 or 1:500 dilution of the pre-BEA PCR was hybridized to 1.5–2 × 10^6^ beads covered with the dual biotinylated primer R-ACH-bead primer (MWG/Operon) [bioteg][bio-on]CAGAGCGTCACAGCCGCCACCACCAGGATGAACAGGAAG, in 1× Titanium buffer and 16 mm MgCl_2_. The last 20 bp of this primer are complementary to the 3′ end of the pre-BEA PCR product. Hybridizations were carried out for 2 min at 94°C, followed by 15 min at 68°C and cooling down to room temperature. The beads were washed once with water and mixed with the aqueous PCR phase (1× Titanium buffer, 8 mm MgCl_2_, 1 mm dNTPs, 9 µm F-ACH 88-GAGCTGGTGGAGGCTGACGA and 50 nm R-93 -CCACCACCAGGATGAACAGGAAG) and the oil phase prepared as described previously (26,28). The emulsion was prepared by vortexing at full speed for 70 s with a 5 mm steel bead (Qiagen) and amplified by an initial denaturation step of 94°C for 2 min, followed by 55 cycles at 94°C for 15 s and 68°C for 75 s with an end step of at 68°C for 2 min. Beads were washed and labeled as described previously (26,28) with wt revA488(/5Alex488N/CAGGCATCCTCAGC*T*A*C*G) and ACH-rev A647 (/5Alex647N/CAGGCATCCTCAGC*T*A*C*G) probes using an initial denaturation step of 95°C for 2 min, followed by 60°C for 5 min, 72°C for 5 min, and kept at 75°C to wash off unextended probes (asterisks denote phosphorothioate bonds).

In each experiment, only ∼10% of the beads carried amplified a product given the low input ratio of DNA molecules to beads assuring that most of the beads get attached to only one initial molecule based on Poisson distribution. Experiments with >30% positives were discarded and repeated, considering that beads seeded with more than one molecule could result in an underestimate of the mutation number. Of the 192 pieces, the average percent positives was ∼8% based on an average of 125 000 beads with a product. Any multicolored bead reflects the presence of both mutant and wild-type molecules due to *de novo* mutations during the BEA and therefore was eliminated during the analysis.

#### Dye switches

After scanning the beads with a microscope as described previously (26,28), the probes were washed off and new probes with reverse labeling wt rev-A647(/5Alex647N/CAGGCATCCTCAGC*T*A*C*G) and ACH-rev A488 (/5Alex488N/CAGGCATCCTCA GC*T*A*C*G) were used to re-label the beads at 95°C for 2 min followed by 63°C for 5 min, 72°C for 5 min and kept at 75°C to wash off unextended probes. The dye switch was performed in a hybridization chamber and *in situ* PCR block as described previously (26). Since beads were immobilized on a polyacrylamide array, it was possible to re-scan the beads without losing the positional information of each bead.

### Bead-calling

For each experiment two sets with three images each were analyzed. Brightfield images between scans (normal scan and dye switch scan) were aligned by computing the normalized cross-correlation matrix of pairs of images in Matlab (Mathworks) as described previously (26). A mask constructed from the combination of both bright fields was employed to extract the intensity values for defined regions of interests (ROI) representing each bead across the fluorescent channels (two per scan). The average pixel intensities for each ROI was compiled for each fluorescent channel to classify the beads into different clusters (10, 01, 11, 00) based on the signal intensity for each fluorophore (the 11 cluster represents beads fluorescing in both channels and the 00 cluster represents empty beads with no fluorescence). The classification scheme was based on a Gaussian Mixture model and normalization parameters were described in detail previously (26). Beads classified as 1001 were counted as wild type and 0110 beads were counted as mutants.

### Control experiments

For experiments used to determine the false-positive mutation frequency of the assay, 1 µg of *E. coli* DNA was spiked with 330 000 copies (equivalent to the amount of FGFR3 copies found in 1 µg of human DNA) of a linearized plasmid with a 550 bp FGFR3 insert containing the wild-type c.1138 locus and amplified by ‘pre-BEA PCR’ (using the same conditions as used for 1 µg of genomic testis DNA). A dilution was further analyzed with BEA as described earlier. The plasmid was constructed by cloning a PCR product derived from blood genomic DNA amplified for 35 cycles using the ‘Pre-BEA PCR’ conditions into a pJET1.2/blunt Cloning Vector (Fermentas) of the CloneJet PCR Cloning Kit (Fermentas). Clones with an insert were verified by PCR and sequencing. The purified plasmid was linearized by digestion with *Xho*I or sonication for 5 s and quantified spectrophotometrically and by qPCR using human genomic DNA as standards. We also confirmed that the amplification efficiency of the plasmid was equivalent to 330 000 human white blood cell genomes.

In order to examine the efficiency of detecting the ACH mutation, different ratios of mutant to wild-type were constructed and processed by the assay (Pre-BEA PCR and BEA using the same conditions as for the testis DNA). For mutant to wild-type FGFR3 ratios of 1:10, 1:100 or 1:1000, triplicate samples contained 1 µg of human blood genomic DNA (Clontech) and varying amounts of genomic DNA derived from an achondroplasia patient (Coriell Cell Repositories). For the 1:10 000 ratio, we measured two samples which contained genomic DNA derived from an achondroplasia patient (30 genomes) and 1 µg of *E. coli* DNA spiked with 330 000 copies of wild-type plasmid (described earlier).

### Estimation of mutation frequencies

The ACH mutation frequency for each testis piece was estimated by the maximum likelihood estimate assuming a Poisson distribution (e.g. 47). The 95% confidence interval was calculated by the method of approximate pivots assuming a Poisson distribution (47). For those pieces for which there were multiple measurements, the number of mutant and wild-type beads for each measurement were combined into a total number of mutant and wild-type beads, and the mutation frequency was estimated from these total numbers by the maximum likelihood estimate assuming a Poisson distribution (by this method those measurements with more beads contribute more to the combined estimate). The estimated frequencies for all of the pieces and their associated 95% confidence intervals are tabulated in Supplementary Material, Table S4. To compute the average mutation frequency for the entire testis, the mutation frequency for each piece was weighted by the number of genomes in each piece. The total number of genomes per testis piece was estimated previously by real time PCR (19).

### Modeling and simulations

The hot spot and selection models have been proposed previously (19–21). Links to the computer code to simulate these models are in the original references and have been collected at  www.cmb.usc.edu/people/petercal/testis/pind2.html. Our computational strategy to test these models is to find the model parameters that best match the average mutation frequency (Av) measured in the testis. We then simulate the models using these parameters and only consider those simulations with Av within 5% of the Av observed for the testis. We repeat until we have one million such simulations (for each model), and then we test whether the summary statistics measuring clustering (Mx/Av, *F* < 50, N95) in the testis match the values of these summary statistics in the simulations. If the summary statistics for the testis are within the range of summary statistics for the simulations then the model is consistent with the data; conversely, if the summary statistics for the testis are outliers for the range of summary statistics in the simulations then the model is rejected (19–21). We also tested whether pieces with fewer than 100 000 wild-type counts and 0 mutants could bias the data by underestimating the mutation frequency. If these pieces were changed to 1 mutant bead, then 69 pieces were affected. The piece with the largest frequency change went from 0 to 280 mutants per million molecules, the average (Av) went from 122 to 175 pmg, there was no change in Mx, the *F* < 50 fraction went from 76 to 66% and the fraction of the testis necessary to capture 95% of mutants (N95) went from 27 to 41%. These changes in the statistics had no effect on the conclusions about the models.

## SUPPLEMENTARY MATERIAL

Supplementary Material is available at *HMG* online.

## FUNDING

This work was supported by the ‘Austrian Science Fund’ (FWF) (P25525-B13 to I.T-B.); and National Institute of General Medical Sciences of the National Institutes of Health under award number R01GM36745 (N.A. and P.C.). The content is solely the responsibility of the authors and does not necessarily represent the official views of the NIGMS or NIH. Funding to pay the Open Access publication charges for this article was provided by the Austrian Science Fund (FWF).

## Supplementary Material

Supplementary Data
