# Supplementary material for: New evidence for positive selection helps explain the paternal age effect observed in achondroplasia
Source: Hum Mol Genet. 2013 Jun 4;22(20):4117–26. doi: 10.1093/hmg/ddt260 (PMC3781639; doi:10.1093/hmg/ddt260)
Supplement: Supplementary Data [file supp_22_20_4117__index.html]

New evidence for positive selection helps explain the paternal age effect observed in achondroplasia — New evidence for positive selection helps explain the paternal age effect observed in achondroplasia — Supplementary Data 

# New evidence for positive selection helps explain the paternal age effect observed in achondroplasia

## 

Supplementary Data

**Files in this Data Supplement:**

- Supplementary Table 1 - xlsx file
- Supplementary Table 2 - xlsx file
- Supplementary Table 3 - xlsx file
- Supplementary Table 4 - xlsx file
